# Circumferential wires as a supplement to intramedullary nailing in unstable trochanteric hip fractures: 4 reoperations in 60 patients followed for 1 year

**DOI:** 10.3109/17453674.2012.665329

**Published:** 2012-06-04

**Authors:** Ilija Ban, Lasse Birkelund, Henrik Palm, Michael Brix, Anders Troelsen

**Affiliations:** ^1^Clinical Orthopaedic Research, Department of Orthopaedics, Hvidovre University Hospital, Copenhagen; ^2^Department of Orthopaedics, Aabenraa Hospital, Aabenraa; ^3^Department of Orthopaedics, Odense University Hospital, Odense, Denmark

## Abstract

**Background and purpose:**

Fixation of unstable trochanteric fractures is challenging. Application of a circumferential wire may facilitate bone contact and avoid postoperative fracture displacement. However, the use of circumferential wires remains controversial due to possible disturbance of the blood supply to the underlying bone. We evaluated the results of applied circumferential wires, concentrating mainly on complications and reoperations.

**Patients and methods:**

60 patients with unstable trochanteric fractures and use of circumferential wires (1 or more) and an intramedullary nail were included from 2 centers. We retrospectively assessed complications and reoperation rates within the first postoperative year.

**Results:**

In 37 of the 60 patients, 2 or more circumferential wires were used. Anatomic reduction was achieved in 24 of the patients and a total cortical displacement of ≤ 10 mm was achieved in 26 other patients. 6 of the 43 patients with radiographic audit after 12 weeks sustained a subsequent fracture displacement of more than 5 mm. 4 patients underwent reoperation: 1 due to deep infection, 1 due to technical failure during osteosynthesis, 1 had a screw cut out, and 1 sustained a new fracture following a new fall.

**Interpretation:**

Application of circumferential wires as a supplement to intramedullary nails in unstable trochanteric fractures is an option as it provides good primary reduction which, in most patients, is maintained over time—with no apparent increase in reoperation rate. Based on our results and on other reports, the use of circumferential wires does not appear to be harmful as sometimes claimed.

Fixation of unstable trochanteric fractures is challenging (Figure 1). Use of a circumferential wire may facilitate bone contact and avoid postoperative fracture displacement. Due to the fear of compromising the blood supply to the underlying bone, surgeons have traditionally been restrictive in using circumferential wires despite the fact that there is little evidence in the literature to support such a detrimental effect. The controversy of circumferential wires probably originates from the work of Charnley et al. (1961) and Newton et al. (1974), who stated that circumferential wires “devitalize bone fragments and prevent the extension of periosteal callus” and that “circumferential wire appliances generally cause nonunion when used simultaneously with intramedullar fixation”. Experimental studies in animals have since shown that circumferential wires are not detrimental to vascularity and bone healing (Wilson et al. 1985, Kirkby et al. 1991), and the few existing clinical studies have shown good results after use of intramedullar fixation supplied with circumferential wires (Partridge et al. 1982, Fitzgerald et al. 1987).

**Figure 1.**
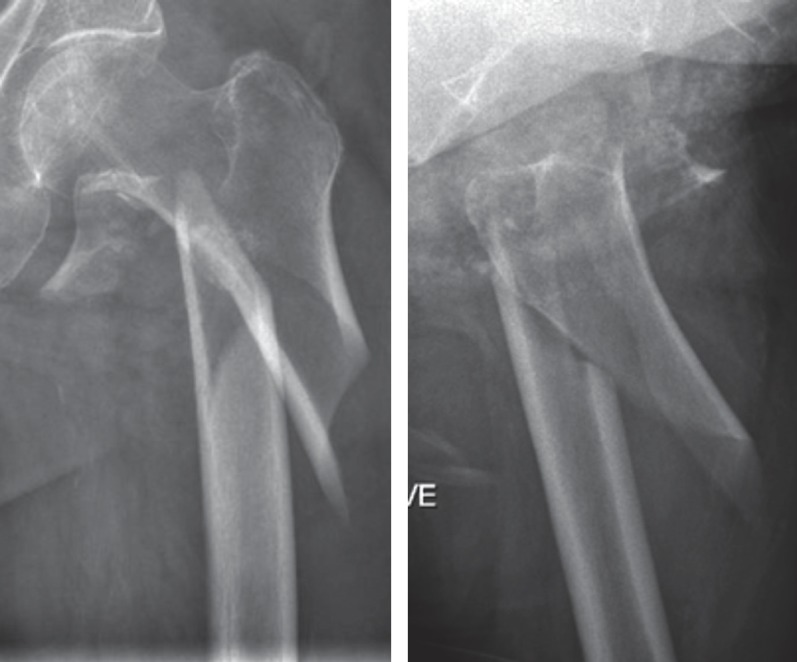
Unstable trochanteric fracture with dislocation.

We evaluated the use of circumferential wires as a supplement to intramedullary nailing in a large cohort of unstable trochanteric hip fractures. Our hypothesis was that if circumferential wires do compromise the blood supply, this would result in an increased risk of bone necrosis and therefore a high rate of infection and failure of fixation.

## Patients and methods

61 consecutive patients with an unstable trochanteric fracture, treated with intramedullary nail and 1 or more circumferential wires, were identified from 2 Danish centers: the University Hospital of Hvidovre, from September 2002 through August 2009, from a total of 2,100 patients with hip fractures; and Aabenraa Hospital, from September 2008 through September 2009, from a total of 403 patients with hip fractures.

1 patient died prior to the first postoperative radiographic control and was excluded from further analysis. Thus, 60 patients (28 from Hvidovre Hospital) were available for analysis. By database inquiry, the following parameters were assessed: patient demographics (age, sex, ASA score, new mobility score (NMS) (0–9)), fracture classification, type of operation and implant, charge of surgeon and supervisor, and whether the patients were allowed to bear full weight immediately postoperatively (Table).

**Table:** Patient characteristics and postoperative fracture position

	n
Total number of patients	60
Demographics	
Females	37
Age (years)	82 (26–100) **^a^**
ASA score III–IV	23
Fracture type (AO classification)	
AO 31 A2 (2.2+2.3)	18
AO 31 A3 (3.1-3.3)	35
AO 32	7
Surgery data	
Duration of surgery (min)	125.5 (60–310) **^a^**
Peroperative bleeding (liters)	0.6 (0.1–3) **^a^**
Postoperative dislocation (mm)	
0	24
1–5	10
6–10	16
> 10	10
Position of implant measured as tip-apex distance (mm)	
0–25	45
> 25	15

**^a^** The values are number of patients except for age, duration of surgery, and intraoperative blood loss which are given as the median with the range in parentheses.

Unstable trochanteric fractures were treated with an intramedullary nail according to the guidelines for treatment of hip fractures at the 2 hospitals (Palm et al. 2012). The decision to add a circumferential wire was based on the individual surgeons’ preferences and was often used in spiral or oblique fractures. The circumferential wires were inserted through a prolonged incision for the collum screw on the lateral side of the thigh. Application of the wire around the femur at the fracture site was done by use of blunt instruments. Additional circumferential wires were applied according to the surgeon’s preferences. Fracture reduction was achieved by tightening the circumferential wires while ensuring correct rotation, length, and anatomic reduction by help of fluoroscopy combined with direct palpation. When fracture reduction was optimal and the circumferential wires were tightened, the nail was inserted through a secondary incision proximal to the greater trochanter. Braided circumferential wires were used in all but four patients. The median operation time was 125 (60–310) min and the median blood loss was 0.6 (0.1–3) L.

Postoperative radiographs and radiographs taken at follow-up during the first year after operation were evaluated retrospectively. Fracture reduction was measured as the sum of cortical displacement perpendicular to the femoral axis in the anterior-posterior and lateral radiographs. Fracture displacement was defined as the difference in fracture position measured immediately postoperatively compared to the last available radiographic follow-up. Implant position was evaluated by measuring the tip-apex distance (Baumgaertner et al. 1995). Postoperative surgical complications (infection, failure of osteosynthesis from all causes, and reoperation for all causes) and mortality within 1 year of the operation were retrospectively assessed by examination of patient files and radiographic material. Radiographic follow-up was not achieved in 17 patients. In 10 cases, the patients were lost to follow-up, and in 7 cases the patient died before follow-up; 15 of the 60 patients died within the first postoperative year.

### Ethics

The data collection was approved by the Danish Data Protection Agency, and the Copenhagen Ethics Committee determined that written patient consent was not required (registration number 2011-41-6030).

## Results

Single circumferential wires were used in 23 patients, with 2 wires used in 28 patients and 3 or more wires in 9 patients. Anatomic reduction was achieved in 24 patients, and in 26 other patients a reduction with an initial total cortical displacement of under 10 mm was achieved (Table). The primary reduction was unchanged in 37 of the 43 patients who were available for radiographic follow-up. 6 of the 43 patients who were available for radiographic follow-up showed subsequent fracture displacement of 5 mm or more. 2 of the 6 patients with a fracture displacement of 5 mm or more were reoperated. In 1 patient, cut-out of the sliding hip screw led to reoperation with a revision arthroplasty, and the other patient underwent reoperation after a fall that resulted in a new fracture at the tip of the nail—which was treated with a plate and additional wires. 2 additional patients were reoperated. 1 needed soft tissue debridement and complete removal of the osteosynthesis because of a deep infection. The last reoperation was due to a collum screw positioned next to the intramedullary nail, and the patient underwent reoperation within a few days with correct placement of the screw. The reoperations were equally distributed (2 at each hospital).

Immediate full weight bearing was allowed in 44 of the 60 cases, and no correlation was found between full weight bearing and increased fracture dislocation or reoperation. Suboptimal initial implant positioning measured by the tip-apex distance (distance > 25 mm) was seen in 15 of the 60 patients. No correlation was found between high tip-apex distance and increased fracture displacement or reoperation.

## Discussion

Our findings indicate that the use of circumferential wires is not detrimental. Within the first year, we found low rates of bone necrosis, infection, and failure of fixation, and the overall reoperation rate of 4/60 in this group of fragile fracture pattern is similar to that in other reports on unstable trochanteric fractures (for review, see Schipper et al. 2004).

**Figure 2.**
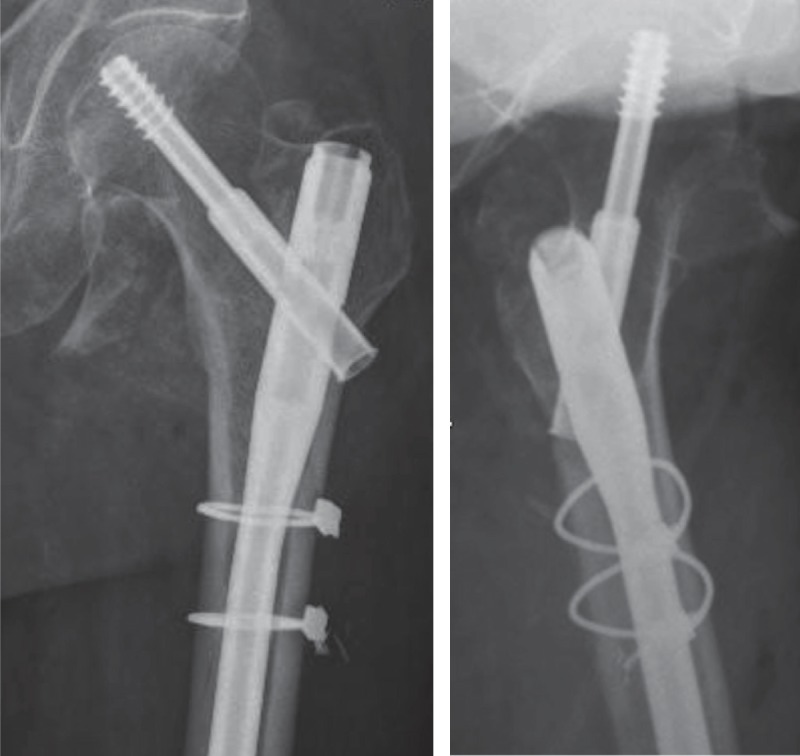
Anatomic reduction of an unstable trochanteric fracture by application of circumferential wires.

Our results also show that circumferential wire fixation appears to facilitate good reduction; anatomic or close to anatomic reduction was achieved in four-fifths of the cases (Figure 2). This is often not technically possible with closed reduction alone. We believe that the good reduction not only eases the nailing procedure but also makes the entire fixation more stable, as most of the weight-bearing forces can be transferred through the aligned osseous fragments. Clamps can also reduce fractures anatomically but a dislocation is often seen after clamp release if a cerclage is not applied (Afsari et al. 2009). Although most of the patients in our study were allowed to bear weight fully immediately after surgery, most reductions obtained primarily were maintained at the follow-up.

The anatomic reduction is obtained at the expense of the additional incision and soft-tissue damage. The operation time and bleeding in our patients were noticeably higher than seen with percutaneous technique (Schipper et al. 2004). This may increase the risk of infection, though this was not seen in the present study (1 of the 60 patients developed deep infection).

The tip-apex distance has been shown to be a reliable predictor of cut-out after sliding hip screw fixation (Pervez et al. 2004). We could not find any correlation between increased tip-apex distance and fixation failure. However, little in known about the importance of the tip-apex distance in predicting failure of fixation after intramedullary nailing.

Since the report by Newton et al. (1974) who found poor results of circumferential wire use in dogs, several animal studies (Wilson et al. 1985, Kirkby et al. 1991) and minor clinical studies (Partridge et al. 1982, Fitzgerald et al. 1987) have not found any correlation between the use of circumferential wires and a higher risk of non-union or infection. Likewise, recent studies have not found the use of modern circumferential wires in subtrochanteric fractures to be harmful (Afsari et al. 2009, Kennedy et al. 2011). These results support ours in that anatomic reduction was achieved in most cases for a more stable construction, which allows early weight bearing.
